# Psychosocial Factors, Smoke-Free Restrictions, and Media Exposure in Relation to Smoking-Related Attitudes and Behaviors among Adults in Armenia and Georgia

**DOI:** 10.3390/ijerph18084013

**Published:** 2021-04-11

**Authors:** Christina N. Wysota, Marina Topuridze, Zhanna Sargsyan, Ana Dekanosidze, Lela Sturua, Michelle C. Kegler, Varduhi Petrosyan, Arusyak Harutyunyan, Varduhi Hayrumyan, Carla J. Berg

**Affiliations:** 1 Department of Prevention and Community Health, Milken Institute School of Public Health, George Washington University, Washington, DC 20052, USA; cwysota@gwu.edu; 2 Non-Communicable Diseases Department, National Center for Disease Control and Public Health, 0198 Tbilisi, Georgia; topuridzemarina@gmail.com (M.T.); ani.dekanosidze@gmail.com (A.D.); lela.sturua@ncdc.ge (L.S.); 3 School of Natural Sciences and Medicine, Ilia State University, 0162 Tbilisi, Georgia; 4 Turpanjian School of Public Health, American University of Armenia, Yerevan 0019, Armenia; zhsargsyan@aua.am (Z.S.); vpetrosi@aua.am (V.P.); aharutyunyan@aua.am (A.H.); vhayrumyan@aua.am (V.H.); 5 Public Health Department, Petre Shotadze Tbilisi Medical Academy, 0144 Tbilisi, Georgia; 6 Department of Behavioral, Social, and Health Education Sciences, Rollins School of Public Health, Emory University, Atlanta, GA 30322, USA; mkegler@emory.edu; 7 Winship Cancer Institute, Emory University, Atlanta, GA 30322, USA; 8 George Washington Cancer Center, George Washington University, Washington, DC 20052, USA

**Keywords:** tobacco control, policy, smoke-free air policy, secondhand smoke exposure, perceived harm, social influences, media exposure

## Abstract

Background: Perceived harm, social influences, smoke-free policies, and media exposure have been understudied in relation to tobacco-related attitudes/behaviors in aggregate or in low and middle-income countries; thus, this study examined these factors collectively in relation to smoking-related outcomes among Armenian and Georgian adults. Methods: Using 2018 cross-sectional survey data (n = 1456), multivariable regression analyses examined these factors in relation to smoking status, perceived harm among nonsmokers, and readiness to quit and past-year quit attempts among smokers. Results: Significant predictors (*p* < 0.05) of current smoking (27.3%) included lower perceived harm, more smoking friends, and fewer home and vehicle restrictions. Among nonsmokers, more home and restaurant/bar restrictions, fewer vehicle restrictions, greater anti-tobacco media exposure, and less pro-tobacco media exposure predicted greater perceived harm. Among smokers, greater perceived social acceptability of smoking, less anti-tobacco media exposure, and greater pro-tobacco media exposure predicted readiness to quit (12.7% of smokers). More smoking friends, more home restrictions, less anti-tobacco media exposure, and greater pro-tobacco media exposure predicted past-year quit attempts (19.2%). Conclusions: Findings support the importance of smoke-free policies but were counterintuitive regarding the roles of social and media influences, underscoring the need to better understand how to address these influences, particularly in countries with high smoking rates.

## 1. Introduction

Cigarette smoking remains a leading cause of preventable death, and those living in low-and middle-income countries (LMICs) are at disproportionate risk for tobacco-related diseases and deaths [1]. Armenia and Georgia represent two LMICs with the highest smoking prevalence among men (11th and 6th highest in the world; 52.3% and 57.7%, respectively), albeit lower among women (1.5% and 5.7%, respectively) [2]. In these countries—and globally—differences in tobacco use profiles are seen with regard to sex and developmental phase (e.g., age, establishing careers, marriage, having children) [1,2].

In countries with high smoking prevalence, it is particularly crucial to identify risk factors for smoking and—among smokers—the likelihood of continuing to smoke. With regard to the latter, most behavior theories (e.g., social cognitive theory, theory of planned behavior, health belief model) [3,4,5,6,7,8,9] emphasize the importance of motivation (or readiness) to quit and self-efficacy (or confidence in one’s ability) to quit as key drivers of cessation attempts and successful cessation. Moreover, determinants of smoking and the likelihood of continued smoking among smokers include perceived risk, social influences, exposure to constraining environments (e.g., places with smoke-free policies), and tobacco-related media exposure. To elaborate, risk perceptions (including perceptions of harm to health) have been shown to predict behavior [10], including smoking [11] and cessation [12] across populations [13]. 

Regarding social influences, social norms theory suggests that perceptions about the beliefs and behaviors of others may influence personal attitude and substance use [14,15]. Injunctive norms are beliefs that others approve or positively appraise a behavior, while descriptive norms are beliefs that others engage in a behavior [14]. Previous studies have found that both injunctive and descriptive norms are associated with increased tobacco use across populations [16]. Thus, key targets for smoking prevention and cessation among those living in LMIC’s may include social norms. 

A key tobacco control strategy globally is implementing smoke-free policies [17,18]. Secondhand smoke exposure (SHSe) poses an additional risk to both smokers and nonsmokers [1] and is particularly prominent in LMICs [19,20]. The recognition of SHSe as a public health risk and the implementation of smoke-free air policies have increased over the years, further driving social influences toward smoking prevention, reduction, and cessation—both in private spaces (e.g., homes, cars) and in public spaces [21]. 

In addition, media communications—both by tobacco control efforts and by the tobacco industry—play a key role in shaping tobacco-related knowledge, opinions, attitudes, and behaviors among individuals and within communities [18,22]. With regard to tobacco control, mass media campaigns are a critical part of comprehensive tobacco control programs in educating about the harms of smoking and SHSe, changing smoking attitudes and social norms, increasing quitting intentions and quit attempts among smokers, reducing overall smoking prevalence, and driving policy change [23]. Key industry strategies include advertising, promotion, and sponsorship, among others [22]. News coverage and public relations may involve either (or both) pro- and anti-tobacco media [22]. Within this context, individuals are differentially impacted by such media for various reasons, including one’s own characteristics (e.g., behavior, perceived risk, and social norms) and how the information is conveyed [24]. 

Despite the broad recognition of these sociocontextual influences on tobacco use, limited research has examined the aggregate influence of perceived risk, social influences, smoke-free policies, and tobacco-related media exposure on smoking behavior, and cessation-related factors among smokers [25], particularly those living in LMICs with large disparities in smoking prevalence between men and women, as in Armenia and Georgia. Moreover, these countries have shown progressive change with respect to tobacco control. The World Health Organization (WHO) Framework Convention on Tobacco Control (FCTC) was ratified in 2004 and 2006 in Armenia and Georgia, respectively; however, few FCTC-recommended policies had been implemented until recently. For example, regarding smoke-free air policies, in 2004, Armenia banned tobacco use in educational, cultural, healthcare, public transportation, and other public settings (except cafes/restaurants) [26], which was extended in 2020 to apply to alternative tobacco products (e.g., e-cigarettes, hookah) and to all public places (including cafes/restaurants) by 2023. In Georgia, new progressive tobacco control laws were implemented in 2017–2018, including a comprehensive smoke-free air policy that similarly covers all alternative tobacco products across a broad range of indoor and outdoor areas. 

Given the gaps in the literature and the nuances of the tobacco-related environments in Armenia and Georgia, the current study examined tobacco use behaviors and attitudes among Armenian and Georgian adults in relation to sociocontextual factors, specifically perceived harm of tobacco use; social influences (e.g., both injunctive and descriptive norms); smoke-free policies in private spaces (i.e., home, car), at the workplace, and in the community (i.e., restaurants, bars); and tobacco-related media exposure (both anti-tobacco and pro-tobacco). We hypothesized that greater perceived harm of tobacco use, fewer social influences who use or support tobacco, more restrictive environments, more anti-tobacco media exposure, and less pro-tobacco media exposure would predict the lower likelihood of tobacco use, less likelihood of future use among nonsmokers, and better indicators of cessation among smokers (e.g., more recent quit attempts, greater readiness, motivation, confidence to quit).

## 2. Materials and Methods

### 2.1. Ongoing Study Overview

This study used a matched-pairs community-randomized controlled trial to examine the effectiveness of local coalitions in promoting smoke-free air among 28 communities in Armenia and Georgia (matched and randomized by key characteristics, e.g., population). The ongoing parent study is more fully described elsewhere [27,28]. 

### 2.2. Data Collection

Among all 28 intervention and control communities, population-level surveys (i.e., of community members) were conducted before the launch of the coalition member training (October–November 2018) [28] and will then be conducted at the culmination of coalition activity. Current analyses focused on baseline population-level surveys conducted in October–November 2018. We aimed to obtain 50 completed surveys/community and obtained census data for all households within the municipality limits from their national Bureau of Statistics. Sampling strategies were different in the two countries because of the nature of the data available from their national Bureaus of Statistics. In Armenia, household census data are available; however, in Georgia, household data are not available but instead, data on “clusters” (i.e., geographically-defined areas of 150 households) are available. In each household, the Kish method [29] was used to identify target participants, who were approached in person at their homes, provided a study description, taken through informed consent, and administered the survey (~20 min) via electronic tablets.

In Armenia, addresses in each city were randomly ordered; assessments began at the beginning of the list and continued until the target recruitment in each city (n = 50) was reached. Overall, 1128 households were visited, of which 27.4% (n = 309) were ineligible (9.3% no household member eligible, 10.6% closed door/not home/did not live there anymore, 6.6% non-existing address). Among the 819 eligible, 705 (86.1%) participated. 

In Georgia, multistage cluster sampling was used to select study participants. In step 1, 5 clusters per city were identified. In step 2, 15 households per cluster were selected using a random walking method: the total number of households was divided by *15* (assuming ~75% response rate) to determine how many households needed to be skipped before arriving at the next designated household (e.g., if the municipality included 150 households, the data collector would go from the first selected household to the 10th). Overall, 958 households were visited, of which 5.0% (n = 48) were ineligible (no household member reachable or eligible). Among the 910 eligible, 751 (82.5%) participated. 

### 2.3. Ethical Considerations 

This study was approved by the Institutional Review Boards of Emory University (#IRB00097093), the National Academy of Sciences of the Republic of Armenia (#IRB00004079), the American University of Armenia (#AUA-2017-013), and the National Center for Disease Control and Public Health of Georgia (#IRB00002150). Before enrollment in the study and collection of data, all participants completed an informed consent. Potential participants were informed that their participation was strictly voluntary and that, if they agreed to participate, they could decline to answer any question or withdraw from the study at any time. To maintain confidentiality, only verbal consent was provided, and only participant IDs were assigned to the address indicated on the data lists. This linked data were subsequently maintained in a secure server to which only key research staff were allowed access as necessary.

### 2.4. Measures

Dependent Variables. Outcomes of interest were current smoking status, cessation-related attitudes and behaviors among smokers (i.e., readiness to quit, past-year quit attempts, quitting importance and confidence), and perceived harm of tobacco use among nonsmokers. Regarding smoking characteristics, we assessed past 30-day cigarette smoking and created a dichotomous outcome of current (past 30-day) smokers versus nonsmokers. Among past 30-day smokers, we assessed: (1) readiness to quit (e.g., in the next 30 days, in the next 6 months), which was used to create a dichotomous outcome of ready to quit in the next 6 months versus not ready; (2) number of past-year quit attempts, which was used to create a dichotomous outcome of any past-year quit attempt versus none; and (3) importance and confidence in quitting (0 = not at all to 10 = extremely), respectively, which were conceptualized as continuous outcomes. Perceived harm was assessed by asking, “How harmful to your health do you think the use of each of the following tobacco products is, on a scale of 1 = not at all harmful to 7 = extremely harmful: regular cigarettes; large or little cigars; electronic cigarettes or vaporizers; heat-not-burn tobacco, such as IQOS; and hookah, waterpipe, or nargila.” We created a perceived harm index score (continuous variable) by calculating the average rating across items (i.e., products assessed; range: 1–7; Cronbach’s alpha = 0.94). This variable was conceptualized as an outcome among nonsmokers (as a proxy for likelihood of use), as well as a predictor for all other outcomes.

Independent Variables. Predictors of interest included other tobacco use characteristics, social influences, smoking restrictions, and tobacco-related media exposure. To further characterize tobacco use behaviors among past 30-day smokers, we assessed the number of days smoked (categorized as every day vs. some days) and cigarettes smoked per day (CPD). 

To assess social influences, participants were asked, “How many of your closest friends (who might include relatives and co-workers) smoke cigarettes? 0 = none; 1 = almost none; 2 = less than half; 3 = about half; 4 = more than half; 5 = almost all; 6 = all.” This item was operationalized as a continuous variable for analysis (range: 0 = 6). We also asked current smokers, “What do people who are important to you, like your friends and family, think about you smoking cigarettes?” and “What do you think the general public’s attitude is towards smoking cigarettes?” with response options of: “0 = all or nearly all disapprove; 1 = most disapprove; 2 = about half approve and half disapprove; 3 = most approve; 4 = all or nearly all approve.” These two items were operationalized as a friend/family/public attitude index score by calculating the average rating across items (range: 0–4; Cronbach’s alpha = 0.59). 

Regarding smoking restrictions, we assessed restrictions in personal settings by asking, “Which of the following statements best describes the smoking rules in your home: smoking in your home is allowed, smoking in your home is generally not allowed with certain exceptions, smoking in your home is never allowed, or there are no rules about smoking in your home? allowed; not allowed but with exceptions; never allowed; no rules.” To assess restrictions in cars, participants were asked, “Which statement best describes the rules about smoking in your household vehicles (cars or trucks)? allowed in all vehicles; sometimes allowed in some vehicles; never allowed in any vehicle; no rules about smoking in the vehicles; don’t own a vehicle.” For each of these items, we created a 3-level restrictions “dose” variable (0 = allowed/no rules, 1 = partial restrictions, 2 = complete restrictions). We recoded “don’t own a vehicle” (n = 790) as “allowed/no rules”, as this represents the lack of a setting with smoking restrictions. 

To assess smoke-free restrictions at work, we first asked participants if they worked outside of the home, and if so, whether their workplace included an indoor setting. Among those indicating that their workplace included an indoor setting, we asked, “Which of the following best describes the policy regarding smoking in indoor areas at your work: smoking is permitted everywhere, smoking is permitted only in certain indoor areas, smoking prohibited in all indoor areas, or there is no policy? permitted everywhere; permitted only in certain indoor areas; prohibited in all indoor areas; there is no policy.” We created a 3-level restriction “dose” variable (0 = allowed/no rules, 1 = partial restrictions, 2 = complete restrictions. We recoded those who were unemployed (n = 743) or employed without indoor settings (n = 31) as “allowed/no rules”, as this represents the lack of a setting with smoking restrictions or related social norms.

Regarding public smoke-free policies, country serves as an indicator of what the actual policy context was during survey administration, as Armenia had limited public smoke-free policies, which did not include restaurants and bars, and Georgia had complete smoke-free policies, which did include restaurants and bars. However, enforcement of and compliance with these policies have been challenges historically [19,30]. Thus, we assessed participants’ perceptions about restrictions in restaurants and bars in their communities by asking, “Which of the following best describes the rules about smoking in (1) restaurants in the community where you live? and (2) drinking establishments, such as a pub or bar, in the community where you live?” Response options include: smoking is allowed in all indoor areas; smoking is allowed only in some indoor areas; smoking is not allowed in any indoor area; every [restaurant/bar] has its own rules.” (Note that the percent reporting complete restaurant restrictions in each country were 5.0% and 77.2% in Armenia and Georgia, respectively, indicating that community members’ experiences did not align fully with actual policy context.) Each of these items was converted to single 3-level restrictions “dose” variables (0 = allowed/no rules, 1 = partial restrictions/each has its own rules, 2 = complete restrictions). We then created a single 3-level restriction for both restaurants and bars (Cronbach’s alpha = 0.94). 

To assess media exposure, participants were asked, “In the past 6 months, how often have you noticed (e.g., on the internet, in social media, in newspapers, in magazines, on TV, on the radio, on signs, or in leaflets): (1) information about the dangers of smoking cigarettes or information that encourages quitting smoking? (2) information about the dangers of being exposed to the smoke of others? (3) any signs in public places indicating that “no smoking is allowed”? (4) any news stories talking about the harms of secondhand smoke or the importance of public smoke-free air policies in your community? (5) any advertisements or signs promoting cigarettes? and (6) any news stories talking about the negative aspects of public smoke-free air policies?” Response options were: 0 = never, 1 = rarely, 2 = sometimes, 3 = frequently. The first 4 questions were summarized as an index (i.e., average) of anti-tobacco media exposure (Cronbach’s alpha = 0.72); the last 2 were summarized as an index score (i.e., average) of pro-tobacco media exposure (Cronbach’s alpha = 0.32; ranges for both: 0–3).

Covariates. Covariates included sociodemographics, specifically age, sex, education level, employment status, monthly household income, marital status, and children under the age of 18 in the home.

### 2.5. Data Analysis 

We first conducted descriptive analyses to characterize participants. Then, we conducted bivariate analyses to examine differences in sociodemographics, smoking-related characteristics, and our primary predictors of interest (i.e., perceived harm, social influences, smoke-free restrictions, media exposure) to the dependent outcomes of: (1) between smokers and nonsmokers; (2) among smokers, in relation to (a) readiness to quit in the next 6 months, (b) past-year quit attempts, (c) importance of quitting, and (d) confidence in quitting; and (3) among nonsmokers, in relation to perceived harm (excluding perceived harm as predictor). 

We then built 6 multivariable regression models for these 6 comparisons (i.e., binary logistic regression for smoking status and, among smokers, readiness to quit and past-year quit attempts; linear regression for importance and confidence to quit among smokers and perceived harm among nonsmokers). The models included country, sociodemographics (age, sex, employment status), perceived harm, number of friends who smoke, smoking restrictions (in the home, household vehicle, workplace, and restaurants/bars), and media exposure (anti- and pro-tobacco); among smokers, models also included smoking level (every day vs. some days) and friend/family/public attitude toward smoking. (Regression analyses were also conducted using multilevel modeling to account for the hierarchical structure of the data (i.e., participants at the individual level nested in communities) [31,32,33]; all intra-class correlations ranged from 0 to 0.01, and findings were not significantly different. Thus, we chose to present the simpler models accounting for country). Sub-analyses by country were also conducted to identify differences in predictors in multivariable regression analyses. All analyses were conducted in SPSS v. 26 (IBM Corporation, Armonk, NY, USA), and alpha was set at 0.05.

## 3. Results

### 3.1. Participant Characteristics

Participants were an average age of 43.45 years old, 60.5% female, 32.1% with ≥Bachelor’s degree, and 49.0% employed (Table 1). Overall, 27.3% reported smoking on some days or every day. Among smokers, 12.7% indicated that they would quit in the next 6 months (including 4.2% indicating within the next month), and 43.5% of smokers never tried to quit, with only 19.2% reporting a past-year quit attempt. Table 1 also presents data by smoking status and by country to characterize differences across the samples. 

### 3.2. Smoking Status

Multivariable analysis (Table 2) indicated that predictors of being a current smoker included: residing in Georgia (vs. Armenia; *p* = 0.015), being male (*p* < 0.001), being employed (vs. unemployed/other; *p* < 0.001), lower perceived harm (*p* < 0.001), more friends who smoke (*p* < 0.001), and fewer smoke-free home and vehicle restrictions (*p* < 0.001). (In subanalyses in which multivariable regression models were constructed among Armenian and Georgian participants, respectively, results indicated that predictors of current smoking included being male and having more friends who smoke in both countries; among Armenian participants, predictors also included having more restaurant/bar restrictions; among Georgian participants, predictors also included being employed, perceiving less harm, and having fewer home and vehicle restrictions.)

### 3.3. Readiness to Quit among Smokers

Supplementary Table S1 provides bivariate results regarding readiness to quit. Multivariable analysis (Table 2) indicated that predictors of readiness to quit included: being unemployed (vs. employed; *p* = 0.005), smoking some days (vs. every day; *p* = 0.002), more favorable attitudes toward smoking among friends/family/general public (*p* = 0.007), less anti-tobacco media exposure (*p* = 0.001), and greater pro-tobacco media exposure (*p* = 0.015). (Subanalysis by country precluded by small cell sizes of those ready to quit.).

### 3.4. Past-Year Quit Attempts among Smokers

Supplementary Table S1 provides bivariate results regarding past-year quit attempts. Multivariable analysis (Table 2) indicated that, among smokers, predictors of attempting to quit in the past year quit included: residing in Armenia (vs. Georgia; *p* = 0.047), younger age (*p* = 0.019), smoking some days (vs. every day; *p* = 0.001), more friends who smoke (*p* = 0.035), more smoke-free home restrictions (*p* = 0.032), less anti-tobacco media exposure (*p* = 0.036), and greater pro-tobacco media exposure (*p* = 0.012). (Subanalysis by country precluded by small cell sizes of those with past-year quit attempts.)

### 3.5. Importance of Quitting among Smokers

Supplementary Table S2 provides bivariate results regarding the importance of quitting among smokers. In multivariable analyses (Table 3), predictors of greater importance of quitting among smokers included: greater perceived harm (*p* < 0.001), less favorable attitudes toward smoking among friends/family/general public (*p* < 0.001), more smoke-free home restrictions (*p* < 0.001), fewer restaurant/bar restrictions (*p* = 0.027), and greater pro-tobacco media exposure (*p* = 0.018). (In subanalyses by country, predictors of importance included greater perceived harm, more negative attitudes toward smoking among others, and more home restrictions in both countries; among Armenian participants, additional predictors included being younger, being female, and fewer restaurant/bar restrictions).

### 3.6. Confidence to Quit among Smokers

Supplementary Table S2 provides bivariate results regarding confidence in quitting. In multivariable analyses (Table 3), predictors of greater confidence in quitting included: being younger (*p* = 0.002), smoking some days (vs. every day; *p* = 0.001), greater perceived harm (*p* = 0.020), more favorable attitudes toward smoking among friends/family/public (*p* = 0.046), and more smoke-free home restrictions (*p* = 0.022). (In subanalyses by country, predictors of confidence among Armenians included smoking some days (vs. every) and among Georgians included younger age, greater perceived harm, more positive attitudes toward smoking among others, and more home restrictions.) 

### 3.7. Perceived Harm among Nonsmokers

Supplementary Table S2 provides bivariate results regarding perceived harm among nonsmokers. Multivariable analysis (Table 3) indicated that predictors of greater perceived harm to health among nonsmokers included: being male (*p* = 0.010), more smoke-free restrictions in the home (*p* = 0.028) and restaurants/bars (*p* = 0.002) but fewer in cars (*p* = 0.026), greater anti-tobacco media exposure (*p* = 0.001), and less pro-tobacco media exposure (*p* < 0.001). (Subanalyses by country found that predictors of greater perceived harm among nonsmokers included being male and having more restaurant/bar restrictions in both countries; in Armenia, predictors also included greater anti-tobacco media exposure and less pro-tobacco media exposure; in Georgia, predictors also included more restaurant/bar restrictions.)

## 4. Discussion

Data from this study sample reflect the smoking prevalence among men and women per national estimates (albeit higher among men; 64% and 3% vs. 52–58% and 2–6%, respectively) [2] and similar cessation-related attitudes as documented in prior studies in other relevant countries/regions (i.e., only ~4% ready to quit in the next month among smokers in LMICs [34], less than half with lifetime quit attempts in European countries [35]). Moreover, these results indicate that key theoretical constructs involved across health behavior theories [3,4,5,6,7,8,9], particularly perceived risk, social influences, and restrictive environments, are critical in shaping—and potentially changing—behavior. Specific to these findings, in general, greater perceived harm of tobacco use, social norms against smoking, and more smoke-free restrictions across settings, particularly in the home, were associated with less likelihood of smoking and with more positive cessation-related behaviors and attitudes among smokers in Armenia and Georgia. However, these findings across outcomes differed, and findings regarding the roles of exposure to anti- and pro-tobacco media exposure are complex. 

As expected, greater perceived harm was associated with being a nonsmoker, and among smokers, with greater cessation-related importance and confidence [10,11,12]. Moreover, it was anticipated that having more friends who smoke was associated with being a current smoker, and perceiving less favorable attitudes toward smoking among friends, family, and the general public was associated with greater importance of quitting among smokers [16,36]. However, other findings regarding social influences were seemingly counterintuitive, both in models across countries and in select subanalysis by country. For example, perceiving more favorable attitudes toward smoking among friends, family, and the general public was associated with readiness to quit and greater confidence in quitting; additionally, having more friends who smoke was associated with making past-year quit attempts. The reasons for these findings are unclear, but they may be due to differing social norms regarding tobacco use in Armenia and Georgia. Given the high smoking prevalence in these countries and the social norms conducive to tobacco use, particularly among men [2], those who have more positive cessation-related behaviors and attitudes may do so despite pro-tobacco social norms within their social networks. Another possibility is that smokers’ may be more likely to perceive that their referent group has a more favorable attitude toward smoking in an effort to justify or rationalize their smoking behavior and, thus, avoid psychological reactance [37]. 

In terms of smoke-free restrictions, having more home smoke-free restrictions was associated with being a nonsmoker and, among smokers, with attempting to quit in the past year, greater importance of quitting, and greater confidence to quit, as has been suggested by prior literature [34,38]. In addition, having more car smoke-free restrictions was associated with being a nonsmoker, and among nonsmokers, more smoke-free home and restaurant/bar restrictions and residing in Georgia (where complete public restrictions were implemented) were associated with greater perceived harm to health among nonsmokers. Unanticipated findings included that greater perceived harm among nonsmokers was associated with fewer restrictions in cars and that greater importance of quitting among smokers was related to fewer restrictions in restaurants/bars. Moreover, country of residence did not reflect what might be expected, as residing in Armenia (where complete public restrictions had not been implemented) was associated with past-year quit attempts and the importance of quitting. The reasons for these findings are unclear; however, there is the possibility that restrictions in primary areas, such as the home and workplace, may influence the extent to which people reserve smoking for cars and that those who perceive quitting as important are more likely to notice or be impacted by smoking around them in public spaces (i.e., the salience heuristic) [39]. 

Regarding media exposure, as expected, among nonsmokers, greater perceived harm to health was associated with greater anti-tobacco media exposure and less pro-tobacco media exposure [34]. However, findings among smokers were all seemingly counterintuitive. For example, we found that less exposure to anti-tobacco media and greater exposure to pro-tobacco media was associated with readiness to quit and having made a past-year quit attempt, and the greater importance of quitting was associated with greater pro-tobacco media exposure among smokers. These findings may be partially explained through psychological reactance and cognitive dissonance experienced by smokers, such that smokers may avoid or dismiss messages that contradict their current behavior [24,39]. The elaboration likelihood model is a theory of persuasion that suggests that there are two different ways people can be persuaded—either peripherally or centrally—depending on how invested they are in a topic [40]. When people hear a message that is personally relevant, for example, they may process information through the central route, in which they carefully consider the information. However, when information may be less relevant or in conflict with one’s beliefs, they may more peripherally process the information. Thus, anti-tobacco messaging may be more noticeable to smokers unlikely to quit and less noticeable to those already motivated to quit; on the other hand, pro-tobacco messaging may be less noticeable to smokers unlikely to quit and more noticeable to those motivated to do so [40].

Other findings indicated that smokers were more likely to be male and employed (as expected [2]). Moreover, those who were younger and/or those smoking less frequently were more likely to be ready to quit and have attempted to quit, and were more confident in their ability to quit. These findings likely reflect cohort effects in countries, such as Armenia and Georgia, where the trends regarding smoking are shifting toward less favorable attitudes toward smoking [41] and that those more motivated to quit are likely to be reducing harm and/or preparing themselves to quit by reducing their overall cigarette consumption [42,43,44]. Comparisons across countries should be interpreted with caution given the high prevalence of smoking among men [2] and the fact that the Armenia sample had a greater proportion of females versus males in their sample. 

These results have implications for research and practice. The counterintuitive findings, particularly regarding perceived social norms and attitudes and media exposure, should be interpreted with caution and lend themselves to other research methods to determine if the associations documented here exist or if cognitive processes are involved in recall and perceptions of these factors. Relatedly, anti-tobacco media messaging must be carefully developed, pilot-tested, and studied over time to determine its actual impact among multiple segments of the nonsmoker and smoker populations, particularly with an eye toward age, sex, and baseline attitudes toward smoking and cessation. In addition, these cross-sectional findings lend themselves to replication using longitudinal approaches to examine potential causality. Finally, multilevel interventions addressing these dynamic multilevel determinants of smoking and cessation in LMICs with distinct sociopolitical contexts regarding smoking are needed.

Limitations. This sample may not represent the general adult populations of these countries. Additionally, the sampling/recruitment methods across countries differed by necessity and yielded different response rates and composition by sex and smoking status. Our results could also be biased due to several factors, such as unmeasured variables associated with differential participation. Finally, the cross-sectional nature and self-reported assessments limit the ability to make causal attributions or account for bias. Thus, these results must be cautiously interpreted.

## 5. Conclusions

Results from this study indicated that several theory-driven factors, particularly perceived harm, social influences, and smoke-free restrictions (especially in the home), are important factors that can reduce adult tobacco use prevalence, either by reducing uptake or by promoting cessation. However, counterintuitive findings regarding social influences and media impact are particularly noteworthy, as they may be indicators of how they are differentially interpreted by subgroups of smokers or of how different sociopolitical contexts might differentially moderate their impact on cessation-related behaviors/attitudes. Ultimately, these findings can help inform multilevel approaches to reducing smoking prevalence in LMICs with high tobacco use rates and shifting tobacco control environments.

## Figures and Tables

**Table 1:** Participant characteristics and bivariate comparisons of nonsmokers vs. smokers and those in Armenia vs. Georgia.

Variable	Total	Nonsmokers	Smokers	*p*	Armenia n = 705	Georgia n = 751	*p*
	n = 1456 (100%)	n = 1058 (72.7%)	n = 398 (27.3%)				
	n (%) or M (SD)	n (%) or M (SD)	n (%) or M (SD)		n (%) or M (SD)	n (%) or M (SD)	
**Country**, n (%)				<0.001			--
Armenia	705 (48.4)	561 (53.0)	144 (36.2)		--	--	
Georgia	751 (51.6)	497 (47.0)	254 (63.8)		--	--	
**Sociodemograhics**							
Age, M (SD)	43.45 (13.49)	43.34 (13.59)	43.38 (13.24)	0.957	42.56 (13.41)	44.08 (13.53)	0.032
Male, n (%)	575 (39.5)	207 (19.6)	368 (92.5)	<0.001	210 (29.8)	365 (48.6)	<0.001
Education, n (%)				0.164			
<High school	233 (15.3)	157 (14.8)	66 (16.6)		161 (22.8)	62 (8.3)	<0.001
High school to some college	765 (52.5)	546 (51.6)	219 (55.0)		327 (45.1)	447 (59.6)	
≥College degree	468 (32.1)	355 (33.6)	113 (28.4)		226 (32.1)	242 (32.2)	
Employed, n (%)	713 (49.0)	438 (41.4)	275 (69.1)	<0.001	311 (44.1)	402 (53.5)	<0.001
Married/cohabitating, n (%)	1061 (72.9)	784 (74.1)	277 (69.6)	0.085	534 (75.7)	527 (70.2)	0.017
Children under 18 in the home, n (%)	731 (51.0)	541 (52.1)	190 (48.1)	0.174	386 (56.6)	345 (45.9)	<0.001
**Smoking Characteristics (for smokers)**							
Number of days smoked, past 30, n (%)				-			0.448
Every day	-	-	350 (87.9)		129 (89.6)	221 (87.0)	
Some days	-	-	48 (12.1)		15 (10.4)	33 (13.0)	
CPD, M (SD)	-	-	22.09 (12.6)	-	21.72 (11.12)	21.02 (10.62)	0.548
Readiness to quit, next 6 months, n (%)			48 (12.7)	-	23 (21.7)	25 (13.7)	0.080
Lifetime quit attempt, n (%)			216 (56.5)	-	100 (74.1)	114 (46.5)	0.001
Past-year quit attempt, n (%)			73 (19.2)	-	42 (29.2)	31 (12.2)	<0.001
Importance of quitting, M (SD)	-	-	5.75 (3.23)	-	6.47 (3.71)	5.33 (2.86)	0.001
Confidence in quitting, M (SD)	-	-	4.80 (3.18)	-	4.78 (3.88)	4.79 (2.73)	0.967
**Perceived Harm**, M (SD)							
Regular cigarettes	5.92 (1.96)	6.14 (1.91)	5.36 (1.97)	<0.001	5.74 (2.18)	6.09 (1.71)	0.001
Large or little cigars	5.83 (1.98)	6.10 (1.87)	5.14 (2.07)	<0.001	5.69 (2.12)	5.97 (1.82)	0.007
Electronic cigarettes or vaporizers	5.33 (2.09)	5.66 (1.96)	4.47 (2.21)	<0.001	5.32 (2.13)	5.35 (2.07)	0.793
Heat-not-burn (IQOS)	5.23 (2.11)	5.55 (1.97)	4.37 (2.21)	<0.001	5.20 (2.14)	5.25 (2.08)	0.644
Hookah/waterpipe/nargila	4.91 (2.22)	5.27 (2.11)	3.96 (2.23)	<0.001	4.78 (2.29)	5.04 (2.15)	0.026
Perceived harm score, M (SD)	5.45 (1.86)	5.74 (1.77)	4.66 (1.87)	<0.001	5.35 (1.92)	5.54 (1.80)	0.047
**Social Influences**							
Number of friends who smoke, n (%)				<0.001			<0.001
None	143 (9.8)	143 (13.5)	0 (0.0)		28 (4.0)	115 (15.5)	
Almost none	175 (12.0)	169 (16.0)	6 (1.5)		84 (12.0)	91 (12.3)	
Less than half	390 (26.8)	310 (29.3)	80 (20.1)		158 (22.6)	232 (31.3)	
About half/Don’t know	324 (22.3)	190 (18.0)	134 (33.7)		170 (24.3)	139 (18.7)	
More than half	310 (21.3)	178 (16.8)	132 (33.2)		166 (23.7)	144 (19.4)	
Almost all	99 (6.8)	61 (5.8)	38 (9.6)		78 (11.2)	21 (2.8)	
All	15 (1.03)	7 (0.7)	8 (2.0)		15 (2.1)	0 (0.0)	
Number friends who smoke score, M (SD)	2.58 (1.43)	2.29 (1.45)	3.35 (1.02)	<0.001	2.94 (1.39)	2.23 (1.38)	<0.001
Friends/family attitude of smoking, n (%)				-			<0.001
All or nearly all disapprove	-	-	105 (27.5)		59 (43.1)	46 (18.8)	
Most disapprove	-	-	181 (47.4)		49 (35.8)	132 (53.9)	
About half approve, half disapprove	-	-	81 (21.2)		24 (17.5)	57 (23.3)	
Most approve	-	-	13 (3.4)		4 (2.9)	9 (3.7)	
All or nearly all approve	-	-	2 (0.5)		1 (0.7)	1 (0.4)	
General public attitude of smoking, n (%)				-			0.001
All or nearly all disapprove	-	-	100 (26.2)		52 (38.0)	48 (19.6)	
Most disapprove	-	-	125 (32.7)		38 (27.7)	87 (35.5)	
About half approve, half disapprove	-	-	148 (38.7)		41 (29.9)	107 (43.7)	
Most approve	-	-	3 (0.9)		2 (1.5)	1 (0.4)	
All or nearly all approve	-	-	6 (1.6)		4 (2.9)	2 (0.8)	
Friend/family/public attitude, M (SD)	*-*	*-*	1.10 (0.72)	-	0.93 (0.83)	1.20 (0.63)	<0.001
**Smoke-free Restrictions**							
Home restrictions, n (%)				<0.001			<0.001
Allowed/no rules	507 (34.9)	348 (32.9)	159 (40.1)		381 (54.2)	126 (16.8)	
Partial restrictions	386 (26.6)	250 (23.7)	136 (34.3)		154 (21.9)	232 (30.9)	
Complete restrictions	561 (38.6)	459 (43.4)	102 (25.7)		168 (23.9)	393 (52.3)	
Dose, M (SD)	1.04 (0.86)	1.11 (0.87)	0.86 (0.80)	<0.001	0.93 (0.83)	1.20 (0.63)	<0.001
Household vehicle restrictions, n (%)				<0.001			<0.001
Allowed/no rules/no car	1044 (71.8)	728 (68.9)	316 (79.6)		533 (75.8)	511 (68.0)	
Partial restrictions	120 (8.3)	73 (6.9)	47 (11.8)		32 (4.6)	88 (11.7)	
Complete restrictions	290 (19.9)	256 (24.2)	34 (27.3)		138 (19.6)	152 (20.2)	
Dose, M (SD)	0.48 (0.81)	0.55 (0.86)	0.29 (0.61)	<0.001	0.44 (0.80)	0.52 (0.81)	0.047
Workplace smoking restrictions, n (%)				<0.001			0.002
Allowed/unemployed/no indoors	987 (67.8)	724 (68.4)	263 (66.1)		505 (71.6)	482 (64.2)	
Partial restrictions	79 (12.0)	41 (10.0)	38 (15.2)		41 (5.8)	38 (5.1)	
Complete restrictions	390 (59.0)	293 (71.3)	97 (38.8)		159 (22.6)	231 (30.8)	
Dose, M (SD)	0.59 (0.88)	0.60 (0.89)	0.58 (0.86)	0.852	0.51 (0.84)	0.67 (0.92)	0.001
Restaurants in your community, n (%)				<0.001			<0.001
Allowed/no rules/don’t know	391 (26.9)	321 (30.5)	70 (17.6)		277 (39.5)	114 (15.2)	
Partial restrictions/each has own rules	441 (30.4)	331 (31.4)	110 (27.7)		389 (55.5)	52 (6.9)	
Complete restrictions	619 (42.7)	402 (38.1)	217 (54.7)		35 (5.0)	584 (77.9)	
Dose, M (SD)	1.16 (0.82)	1.08 (0.83)	1.37 (0.77)	<0.001	0.65 (0.57)	1.63 (0.73)	<0.001
Bars in your community, n (%)				<0.001			<0.001
Allowed/no rules	488 (33.6)	396 (37.6)	92 (23.2)		341 (48.6)	147 (19.6)	
Partial restrictions/each has own rules	371 (25.6)	280 (26.6)	91 (22.9)		334 (47.6)	37 (4.9)	
Complete restrictions	592 (40.8)	378 (35.9)	214 (53.9)		26 (3.7)	566 (75.5)	
Dose, M (SD)	1.07 (0.86)	0.98 (0.86)	1.31 (0.82)	<0.001	0.55 (0.57)	1.56 (0.80)	<0.001
Restaurant/bar restrictions dose, M (SD)	2.23 (1.63)	2.06 (1.63)	2.68 (1.54)	<0.001	1.21 (1.05)	3.19 (1.49)	<0.001
**Media Exposure, M (SD)**							
Anti-tobacco media							
Dangers of smoking cigarettes	1.84 (1.14)	1.89 (1.12)	1.69 (1.19)	0.003	1.92 (1.09)	1.76 (1.19)	0.010
Dangers of SHSe	1.50 (1.20)	1.55 (1.18)	1.36 (1.23)	0.007	1.54 (1.15)	1.46 (1.24)	0.190
“No smoking is allowed” sign	2.27 (0.99)	2.23 (1.00)	2.38 (0.95)	0.012	1.99 (1.01)	2.54 (0.89)	<0.001
News on SHSe	0.69 (0.98)	0.71 (0.98)	0.64 (0.99)	0.126	0.66 (0.90)	0.72 (1.05)	0.264
Anti-tobacco media exposure score	1.58 (0.80)	1.60 (0.80)	1.52 (0.81)	0.094	1.53 (0.75)	1.62 (0.85)	0.031
Pro-tobacco media							
Advertisements promoting cigarettes	0.31 (0.73)	0.33 (0.76)	0.24 (0.64)	0.030	0.40 (0.84)	0.22 (0.60)	<0.001
Anti-smoke-free policy media	0.69 (0.98)	0.55 (0.92)	0.45 (0.85)	0.073	0.65 (0.96)	0.40 (0.84)	<0.001
Pro-tobacco media exposure score	0.42 (0.64)	0.44 (0.66)	0.35 (0.57)	0.012	0.53 (0.72)	0.31 (0.52)	<0.001

Notes: M = Mean; SD = Standard Deviation; n = Number; *p* = *p*-value. M and SD reported to hundredths; % reported to tenths; *p*-values reported to thousandths.

**Table 2:** Binary logistic regression analyses examining predictors of smoking status among all participants and readiness to quit in the next 6 months and past-year quit attempt among past 30-day smokers.

Variable	Among All Participants			Among Smokers					
	Smoker			Readiness to Quit			Past-Year Quit Attempt		
	OR	CI	*p*	OR	CI	*p*	OR	CI	*p*
**Country: Georgia (ref: Armenia)**	1.96	1.14, 3.36	0.015	0.74	0.28, 1.96	0.549	0.39	0.15, 0.99	0.047
**Sociodemograhic and Smoking Factors**									
Age	1.00	0.99, 1.02	0.557	0.99	0.97, 1.02	0.476	0.97	0.95, 1.00	0.019
Male (ref: female)	38.51	24.12, 61.48	<0.001	3.13	0.66, 14.76	0.149	5.51	0.64, 47.20	0.120
Employed (ref: not employed)	2.13	1.34, 3.37	0.001	0.30	0.13, 0.70	0.005	0.88	0.42, 1.83	0.737
Smoke every day (ref: some days)	--	--	--	0.22	0.09, 0.58	0.002	0.21	0.08, 0.54	0.001
**Perceived Harm Score**	0.77	0.70, 0.85	<0.001	0.93	0.78, 1.12	0.463	1.08	0.91, 1.28	0.366
**Social Influences**									
Number of friends who smoke	1.61	1.37, 1.88	<0.001	0.81	0.57, 1.15	0.244	1.38	1.02, 1.86	0.035
Friend/family/public attitude	--	--	--	0.46	0.26, 0.81	0.007	0.84	0.55, 1.29	0.432
**Smoke-free Restrictions (doses)**									
Home restrictions	0.50	0.36, 0.64	<0.001	1.34	0.85, 2.12	0.209	1.55	1.04, 2.32	0.032
Household vehicle restrictions	0.60	0.47, 0.77	<0.001	0.71	0.39, 1.31	0.274	0.70	0.40, 1.22	0.205
Workplace (indoor) restrictions	0.82	0.64, 1.06	0.829	1.53	0.94, 2.51	0.089	1.28	0.86, 1.91	0.223
Restaurant/bar restrictions	1.14	0.97, 1.34	0.109	1.01	0.74, 1.38	0.960	0.93	0.69, 1.25	0.625
**Media Exposure**									
Anti-tobacco media exposure	1.06	0.83, 1.36	0.625	0.45	0.28, 0.72	0.001	0.64	0.43, 0.97	0.036
Pro-tobacco media exposure	0.76	0.56, 1.04	0.090	2.12	1.16, 3.89	0.015	2.05	1.17, 3.60	0.012
**Nagelkerke R-square**	0.657			0.190			0.246		

Notes: OR = Odds Ratio; CI = 95% Confidence Interval; *p* = *p*-value. OR and CI reported to hundredths; *p*-values reported to thousandths.

**Table 3:** Linear regression analyses examining predictors of the importance of quitting and confidence to quit among past 30-day smokers and perceived harm among nonsmokers.

Variable					Among Smokers				Among Nonsmokers			
	Importance				Confidence				Perceived Harm			
	B	CI	SE	*p*	B	CI	SE	*p*	B	CI	SE	*p*
**Country: Georgia (ref: Armenia)**	−0.13	−1.05, 0.78	0.46	0.772	−0.82	−1.79, 0.14	0.49	0.094	0.08	−0.21, 0.37	0.15	0.609
**Sociodemograhic and Smoking Factors**												
Age	−0.01	−0.03, 0.01	0.01	0.397	−0.04	−0.07, −0.02	0.01	0.002	−0.00	−0.01, 0.01	0.01	0.722
Male (ref: female)	−0.05	−1.31, 1.21	0.64	0.933	−0.10	−1.43, 1.23	0.68	0.883	0.37	0.09, 0.64	0.14	0.010
Employment (ref: other)	−0.15	−0.89, 0.58	0.37	0.680	0.53	−0.24, 1.30	0.39	0.179	−0.15	−0.49, 0.20	0.17	0.405
Smoke every day (ref: some days)	−0.23	−1.23, 0.84	0.54	0.678	−1.83	−2.96, −0.71	0.57	0.001	--	--		--
**Perceived Harm Score**	0.41	0.24, 0.59	0.09	<0.001	0.22	0.03, 0.40	0.09	0.020	--	--		--
**Social Influences**												
Number of friends who smoke	0.05	−0.27, 0.35	0.16	0.777	−0.21	−0.53, 0.12	0.17	0.219	−0.01	−0.09, 0.07	0.04	0.827
Friend/family/public attitude	−1.17	−1.63, −0.72	0.23	<0.001	0.49	0.01, 0.97	0.24	0.046	--	--		--
**Smoke-free Restrictions (doses)**												
Home restrictions	0.79	0.37, 1.21	0.21	<0.001	0.52	0.08, 0.97	0.23	0.022	0.16	0.02, 0.30	0.07	0.028
Household vehicle restrictions	−0.34	−0.89, 0.20	0.28	0.216	−0.25	−0.82, 0.33	0.29	0.402	−0.14	−0.27, −0.02	0.06	0.026
Workplace (indoor) restrictions	0.27	−0.15, 0.68	0.21	0.213	−0.02	−0.46, 0.43	0.23	0.947	0.04	−0.15, 0.22	0.10	0.706
Restaurant/bar restrictions	−0.31	−0.59, −0.04	0.14	0.027	0.07	−0.23, 0.36	0.15	0.679	0.13	0.05, 0.21	0.04	0.002
**Media Exposure**												
Anti-tobacco media exposure	−0.25	−0.67, 0.17	0.21	0.238	0.12	−0.32, 0.57	0.23	0.587	0.26	0.11, 0.40	0.07	0.001
Pro-tobacco media exposure	0.72	0.13, 1.32	0.30	0.018	−0.57	−1.20, 0.07	0.32	0.080	−0.64	−0.81, −0.47	0.09	<0.001
**Adjusted R-square**	0.224				0.087				0.090			

Notes: B = Beta; CI = 95% Confidence Interval; SE = Standard Error; *p* = *p*-value. B, CI, and SE reported to hundredths; *p*-values reported to thousandths.

## Data Availability

Limited data sets available upon request to the corresponding author.

## Supplementary Materials

The following are available online at https://www.mdpi.com/article/10.3390/ijerph18084013/s1, Table S1: Bivariate analyses regarding readiness to quit in the next 6 months and past-year quit attempt among past 30-day smokers, Table S2: Bivariate analyses examining predictors of importance of quitting and confidence to quit among past 30-day smokers and perceived harm among nonsmokers.
