# Supplementary material for: Psychosocial Factors, Smoke-Free Restrictions, and Media Exposure in Relation to Smoking-Related Attitudes and Behaviors among Adults in Armenia and Georgia
Source: Int J Environ Res Public Health. 2021 Apr 11;18(8):4013. doi: 10.3390/ijerph18084013 (PMC8068968; doi:10.3390/ijerph18084013)
Supplement: Supplementary file 1 [file ijerph-18-04013-s001.pdf]

Supplementary Table 1. Bivariate analyses regarding readiness to quit in the next 6 months and past-year quit attempt among past 30-day smokers

	Smokers	Not ready to quit	Ready to quit		No past-year quit attempt	Past-year quit attempt	
	N=377 (100%)	N=329 (87.3%)	N=48 (12.7%)		N=307 (80.8%)	N=73 (19.2%)	
**Variable**	**N (%) or M (SD)**	**N (%) or M (SD)**	**N (%) or M (SD)**	**p**	**N (%) or M (SD)**	**N (%) or M (SD)**	**p**
**Country,** N (%)				.049			<.001
Armenia	136 (36.1)	113 (34.3)	23 (47.9)		93 (30.7)	41 (56.9)	
Georgia	241 (63.9)	216 (65.7)	25 (52.1)		210 (69.3)	31 (43.1)	
**Sociodemograhics**							
Age, M (SD)	43.27 (13.12)	43.40 (13.23)	42.40 (12.45)	.622	44.21 (13.21)	39.07 (12.58)	.003
Male, N (%)	351 (93.1)	306 (93.0)	45 (93.8)	.850	282 (91.9)	72 (98.6)	.039
Education, N (%)				.590			.053
<High school	63 (16.7)	56 (17.0)	7 (14.6)		47 (15.3)	17 (23.3)	
High school to some college	204 (54.1)	180 (54.7)	24 (50.0)		178 (58.0)	30 (41.1)	
≥College degree	110 (29.2)	93 (28.3)	17 (35.4)		82 (26.7)	26 (25.6)	
Employed, N (%)	112 (29.7)	237 (72.0)	28 (58.3)	.052	212 (69.1)	55 (75.3)	.291
Married/cohabitating, N (%)	263 (69.8)	231 (87.8)	32 (12.2)	.617	215 (81.4)	49 (18.6)	.628
Children under 18 in the home, N (%)	182 (48.7)	160 (87.9)	22 (12.1)	.674	162 (83.1)	33 (16.9)	.395
**Smoking Characteristics**							
Smoked every day (vs. some), past 30, N (%)	342 (91.0)	304 (92.7)	38 (79.2)	.002	284 (92.8)	59 (81.9)	.004
CPD, M (SD)	22.2 (12.55)	22.48 (12.38)	20.23 (13.59)	.247	22.47 (12.35)	20.66 (13.52)	.270
Readiness to quit, next 6 months, N (%)	--	--	--	--	27 (8.9)	21 (29.2)	<.001
Past-year quit attempt, N (%)	72 (19.2)	51 (15.6)	21 (43.8)	<.001	--	--	--
Importance of quitting, M (SD)	5.76 (3.24)	5.42 (3.21)	8.10 (2.31)	<.001	5.34 (3.10)	7.38 (3.22)	<.001
Confidence in quitting, M (SD)	4.75 (3.16)	4.58 (3.18)	5.96 (2.77)	.005	4.74 (3.18)	5.04 (3.26)	.464
**Perceived Harm Score,** M (SD)	4.65 (1.87)	4.64 (1.87)	4.69 (1.83)	.855	4.59 (1.87)	4.92 (1.82)	.172
**Social Influences,** M (SD)							
Number of friends who smoke	3.37 (1.03)	3.38 (1.05)	3.29 (0.90)	.579	3.28 (1.02)	3.70 (1.00)	.002
Friend/family/public attitude	1.10 (0.72)	1.14 (0.73)	0.85 (0.59)	.001	1.14 (0.71)	0.97 (0.73)	.064
**Smoke-free Restrictions (doses),** M (SD)							
Home restrictions	0.84 (0.80)	0.82 (0.80)	0.96 (0.77)	.277	0.81 (0.78)	0.96 (0.84)	.162
Household vehicle restrictions	0.29 (0.63)	0.29 (0.62)	0.31 (0.66)	.806	0.29 (0.61)	0.32 (0.64)	.724
Workplace (indoor) restrictions	0.59 (0.86)	0.58 (0.85)	0.67 (0.91)	.502	0.54 (0.84)	0.75 (0.92)	.060
Restaurant/bar restrictions	2.71 (1.54)	2.73 (1.55)	2.56 (1.50)	.484	2.79 (1.54)	2.34 (1.98)	.027
**Media Exposure,** M (SD)							
Anti-tobacco media exposure	1.51 (0.80)	1.54 (0.81)	1.31 (0.73)	.065	1.53 (0.82)	1.45 (0.73)	.434
Pro-tobacco media exposure	0.33 (0.56)	0.31 (0.55)	0.48 (0.63)	.052	0.29 (0.48)	0.53 (0.81)	.001

Notes: M=Mean; SD=Standard Deviation; N=Number; p=p-value. M and SD reported to hundredths; % reported to tenths; p-values reported to thousandths.

Supplementary Table 2. Bivariate analyses examining predictors of importance of quitting and confidence to quit among past 30-day smokers and perceived harm among nonsmokers

	Smokers, N=382				Nonsmokers, N=1,058	
	Importance of quitting		Confidence in quitting		Perceived harm	
**Variable**	**M (SD) or r**	**p**	**M (SD) or r**	**p**	**M (SD) or r**	**p**
**Country,** M (SD)		.001		.940		<.001
Armenia	6.50 (3.69)		4.82 (3.88)		5.49 (1.89)	
Georgia	5.33 (2.86)		4.79 (2.73)		6.03 (1.57)	
**Sociodemograhics**						
Age, r	-0.05	.330	-0.19	<.001	-0.00	.986
Male, M (SD)	5.76 (3.45)	.776	4.81 (3.19)	.857	5.55 (1.69)	.077
Female	5.58 (3.00)		4.69 (3.16)		5.79 (1.78)	
Education, M (SD)		.011		.605		.588
<High school	6.00 (3.81)		4.45 (3.85)		5.62 (1.80)	
High school to some college	5.32 (3.08)		4.83 (3.10)		5.74 (1.78)	
≥College degree	6.43 (3.03)		4.95 (2.92)		5.80 (1.73)	
Employed, M (SD)	5.82 (3.15)	.512	5.01 (3.17)	.043	5.67 (1.82)	.262
Unemployed /Other	5.58 (3.41)		4.29 (3.18)		5.79 (0.73)	
Married/cohabitating, M (SD)	5.96 (3.07)	.053	4.80 (3.18)	.974	5.82 (1.74)	.020
Other	5.27 (3.07		4.79 (3.21)		5.53 (1.83)	
Children under 18 in the home, M (SD)	6.04 (3.28)	.087	4.61 (3.31)	.292	5.78 (1.71)	.440
No	5.47 (3.14)		4.96 (3.04)		5.69 (1.84)	
**Smoking Characteristics**						
Smoke every day, past 30, M (SD)	5.70 (3.29)	.539	4.61 (3.18)	.001		
Some days	6.06 (2.61)		6.54 (2.68)		--	--
CPD, r	-0.15	.003	-0.18	<.001	--	--
Ready to quit, next 6 months, M (SD)	8.10 (2.31)	<.001	6.00 (2.77)	.005		
No	5.42 (3.21)		4.58 (3.18)		--	--
Past-year quit attempt, M (SD)	7.38 (3.22)	<.001	5.04 (3.26)	.464		
No	5.34 (3.10)		4.74 (3.18)		--	--
Importance of quitting, r	--	--	0.14	.007	--	--
Confidence in quitting, r	0.14	.007	--	--	--	--
**Perceived Harm Score,** r	0.34	<.001	0.12	.017	--	--
**Social Influences,** r						
Number of friends who smoke	0.03	.543	-0.07	.165	-0.07	.028
Friend/family/public attitude	-0.38	<.001	0.03	.503	--	--
**Smoke-free Restrictions (doses)**, r						
Home restrictions	0.19	<.001	0.15	.003	0.11	.001
Household vehicle restrictions	0.05	.345	0.03	.615	-0.06	.069
Workplace (indoor) restrictions	0.16	.002	0.08	.101	0.02	.526
Restaurant/bar restrictions	-0.11	.038	0.05	.332	0.16	<.001
**Media Exposure,** r						
Anti-tobacco media exposure	0.05	.359	0.07	.170	-0.22	<.001
Pro-tobacco media exposure	0.11	.025	-0.04	.455	0.05	.084

Notes: M=Mean; SD=Standard Deviation; N=Number; p=p-value. M and SD reported to hundredths; % reported to tenths; p-values reported to thousandths.
